# Cost-effectiveness Analysis of Bariatric Surgery for Patients With Nonalcoholic Steatohepatitis Cirrhosis

**DOI:** 10.1001/jamanetworkopen.2019.0047

**Published:** 2019-02-22

**Authors:** Matthew J. Klebanoff, Kathleen E. Corey, Sumeyye Samur, Jin G. Choi, Lee M. Kaplan, Jagpreet Chhatwal, Chin Hur

**Affiliations:** 1 Currently a medical student at Yale School of Medicine, New Haven, Connecticut; 2 Gastroenterology Division, Massachusetts General Hospital, Boston; 3 Harvard Medical School, Boston, Massachusetts; 4 Institute for Clinical and Economic Review, Boston, Massachusetts; 5 Massachusetts General Hospital Institute for Technology Assessment, Boston; 6 Division of Digestive and Liver Diseases, Department of Medicine,Columbia University Medical Center, New York, New York

## Abstract

**Importance:**

Obesity is the most common risk factor for nonalcoholic steatohepatitis (NASH), the progressive form of nonalcoholic fatty liver disease that can lead to cirrhosis and hepatocellular carcinoma. Weight loss can be an effective treatment for obesity and may slow the progression of advanced liver disease.

**Objective:**

To assess the cost-effectiveness of bariatric surgery in patients with NASH and compensated cirrhosis.

**Design, Setting, and Participants:**

This economic evaluation study used a Markov-based state-transition model to simulate the benefits and risks of laparoscopic sleeve gastrectomy (SG), laparoscopic Roux-en-Y gastric bypass (GB), and intensive lifestyle intervention (ILI) compared with usual care in patients with NASH and compensated cirrhosis and varying baseline weight (overweight, mild obesity, moderate obesity, and severe obesity). Patients faced varied risks of perioperative mortality and complications depending on the type of surgery they underwent. Data were collected on March 22, 2017.

**Main Outcomes and Measures:**

Life-years, quality-adjusted life-years (QALYs), costs (in 2017 $US), and incremental cost-effectiveness ratios (ICERs) were calculated.

**Results:**

Demographic characteristics of the patient population were based on a previously published prospective study (n = 161). Patients in the model were 41.0% female, and the base case age was 54 years. Compared with usual care, SG was associated with an increase in QALYs of 0.263 to 1.180 (bounds of ranges represent overweight to severe obesity); GB, 0.263 to 1.207; and ILI, 0.004 to 0.216. Sleeve gastrectomy was also associated with an increase in life-years of 0.693 to 1.930; GB, 0.694 to 1.947; and ILI, 0.012 to 0.114. With usual care, expected life-years in overweight, mild obesity, moderate obesity, and severe obesity were 12.939, 11.949, 10.976, and 10.095, respectively. With usual care, QALY in overweight was 6.418; mild obesity, 5.790; moderate obesity, 5.186; and severe obesity, 4.577. Sleeve gastrectomy was the most cost-effective option for patients across all weight classes assessed: ICER for SG in patients with overweight was $66 119 per QALY; mild obesity, $18 716 per QALY; moderate obesity, $10 274 per QALY; and severe obesity, $6563 per QALY. A threshold analysis on the procedure cost of GB found that for GB to be cost-effective, the cost of the surgery must be decreased from its baseline value of $28 734 by $4889 for mild obesity, by $3189 for moderate obesity, and by $2289 for severe obesity. In overweight patients, GB involved fewer QALYs than SG, and thus decreasing the cost of surgery would not result in cost-effectiveness.

**Conclusions and Relevance:**

Bariatric surgery could be highly cost-effective in patients with NASH compensated cirrhosis and obesity or overweight. The findings from this analysis suggest that it can inform clinical trials evaluating the effect of bariatric procedures in patients with NASH cirrhosis, including those with a lower body mass index.

## Key Points

**Question:**

Is bariatric surgery cost-effective in patients with nonalcoholic steatohepatitis and compensated cirrhosis?

**Findings:**

In this simulation model study, laparoscopic sleeve gastrectomy had an incremental cost-effectiveness ratio of $66 119 per quality-adjusted life-year in overweight patients, $18 716 per quality-adjusted life-year in patients with mild obesity, $10 274 per quality-adjusted life-year in patients with moderate obesity, and $6563 per quality-adjusted life-year in patients with severe obesity.

**Meaning:**

Bariatric surgery could be highly cost-effective in patients with nonalcoholic steatohepatitis and compensated cirrhosis, even in those with a lower baseline body mass index.

## Teaser

This economic evaluation study uses a simulation model to assess the cost-effectiveness of bariatric surgery in patients with nonalcoholic steatohepatitis (NASH) and compensated cirrhosis who have varying baseline weight.

## Introduction

The prevalence of adult obesity in the United States is expected to reach approximately 50% by 2030.^1^ Obesity is the most common risk factor for nonalcoholic fatty liver disease and the progressive subtype of the disease, nonalcoholic steatohepatitis (NASH), which can give rise to cirrhosis and hepatocellular carcinoma (HCC). In recent prospective studies, more than 70% of patients with compensated cirrhosis were overweight or obese.^2^ In addition to contributing to the development of advanced liver disease, obesity also leads to worse outcomes among patients with compensated cirrhosis. Compared with their normal-weight counterparts, obese patients with compensated cirrhosis face a nearly 3-fold increased risk of decompensation.^2^

Weight loss can be an effective treatment for NASH.^3^ In a recent 12-month trial of lifestyle interventions, fibrosis improved or stabilized in patients with NASH who lost at least 5% of total body weight.^4^ Weight loss through diet and exercise also decreases portal pressure in patients with NASH and compensated cirrhosis, which likely reduces the likelihood of decompensation.^5^ In addition, weight loss may also improve the likelihood of receiving a liver transplant for patients on the wait list.^6^ Although diet and exercise may improve outcomes in patients with NASH, long-term maintenance of weight loss is often inadequate. In a trial of lifestyle intervention in patients with NASH, only 50% achieved 7% total body weight loss after 1 year.^4^

In contrast with lifestyle interventions, bariatric surgery often induces excellent long-term weight loss outcomes and may also have the potential to halt or reverse liver damage in cirrhosis.^7,8^ However, bariatric surgery carries heightened perioperative risks in patients with cirrhosis. An analysis of patients undergoing bariatric surgery in the Nationwide Inpatient Sample showed that mortality was higher in those with compensated (0.9%) and decompensated cirrhosis (16.3%) than in those without cirrhosis (0.3%).^9^ In addition, the cost of bariatric surgery may pose a potential barrier for some patients; payers typically deny coverage of these procedures to individuals with body mass index (BMI; calculated as weight in kilograms divided by height in meters squared) of less than 35.0, making surgery inaccessible to many who might benefit from it.

Despite the benefits of weight loss in NASH cirrhosis and the increasing burden of this condition, no randomized clinical trial has been performed, to date, to assess the effect of bariatric surgery and nonsurgical weight loss interventions on the progression of advanced liver disease. Under these circumstances, mathematical modeling provides a platform to integrate the best available data to help guide medical decision making. The aim of our study was to perform a comparative cost-effectiveness analysis of bariatric surgery in patients with NASH cirrhosis who were overweight or obese.

## Methods

### Model Structure

This report follows the Consolidated Health Economic Evaluation Reporting Standards (CHEERS) reporting guideline for economic evaluations. We developed a Markov-based state-transition model using TreeAge Pro software (version 2017; TreeAge) to assess cost-effectiveness of the following 4 different weight loss strategies: usual care, intensive lifestyle intervention (ILI), laparoscopic sleeve gastrectomy (SG), and laparoscopic Roux-en-Y gastric bypass (GB) (Figure). Institutional review board approval was not required for this study that did not use human participants.

**Figure.**
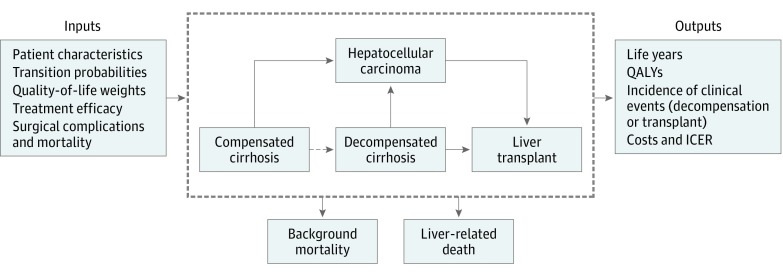
Simplified Model Schematic The dashed line between the compensated cirrhosis and decompensated cirrhosis states indicates that the probability of decompensating varies according to body mass index and thus decreases with weight loss. ICER indicates incremental cost-effectiveness ratio; QALYs, quality-adjusted life-years.

The baseline patient characteristics of the simulated population were based on a previously published prospective study^2^ (n = 161). Patients in the model were 41.0% female, and the base case age was 54 years. Note that this population represents patients with cirrhosis and thus includes a higher proportion of older and male individuals than the overall population of patients undergoing bariatric surgery. Our analysis was conducted for patients with overweight, mild obesity, moderate obesity, and severe obesity. The model cycle length, or time between state transitions, was 1 year. Each year, patients could remain in the same health state, progress to another, or die. Patients with compensated cirrhosis could progress to the decompensated cirrhosis state. Based on an analysis that associated obesity with clinical decompensation,^2^ we adjusted the probability of decompensation according to patients’ BMI. Patients with compensated or decompensated cirrhosis could progress to HCC.^10,11,12,13,14^ In the model, liver transplant was a possibility only for individuals with decompensated cirrhosis or HCC (Figure).^15,16,17,18^ After transplant, these patients faced a risk of death based on mortality rates among transplant recipients.^19^ Possible causes of death included surgical mortality, liver-related mortality, and background mortality, which was based on patients’ age, sex, and BMI, using data from the US Third National Health and Nutritional Examination Survey.^9,20,21,22,23^ Patients with compensated and decompensated cirrhosis had excess liver-related mortality of 2.1% and 13.0%, respectively.^11,12,22^ Simulated patients were followed up until death, to capture the long-term benefits of surgery.

### Competing Strategies

In the usual-care strategy, patients did not undergo surgery or other weight loss interventions, and their liver disease progressed according to probabilities derived from the literature. We assumed that, without intervention, patients’ weight remained constant during their lifetime.

In SG and GB strategies, patients faced a risk of 30-day surgical mortality after the operation, which varied depending on the type of procedure, based on the American College of Surgeons National Quality Improvement Survey.^23^ They also encountered the possibility of major and minor complications after surgery.^23^ Major complications required a second operation; minor complications required readmission but no additional operation. The estimates from the American College of Surgeons National Quality Improvement Survey were adjusted for our population using data from a study that compared mortality in patients with compensated cirrhosis and patients without cirrhosis.^9^

After surgery, patients underwent weight loss, which could slow the progression of liver disease in our model by decreasing the probability of decompensation. We incorporated the association of weight loss with survival and quality of life for patients in the following 4 weight classes: overweight (BMI, 25.0-29.9), mild obesity (BMI, 30.0-34.9), moderate obesity (BMI, 35.0-39.9), and severe obesity (BMI, ≥40.0). Postsurgical weight loss was derived from a recent meta-analysis.^24^ Weight loss in the ILI strategy was derived from the Look AHEAD (Action for Health in Diabetes) trial.^25^ Long-term trends in weight were extrapolated using data from the Swedish Obese Subjects study.^26^ We applied our estimates of percentage of excess weight loss (eTable 1 in the Supplement) to all of the initial weight categories in our analysis.

### Costs and Quality-of-Life Adjustments

Costs associated with treatment strategies, surgical complications, and health states were derived from the literature.^27,28,29,30,31,32^ The initial cost of GB was based on a prior analysis,^27^ and the cost of SG was calculated using cost data from the Healthcare Cost and Utilization Project Nationwide Inpatient Sample.^28^ All costs from prior years were converted to 2017 $US using the Consumer Price Index.^33^ Costs were evaluated from the perspective of a third-party payer.

Utility estimates were derived from studies on cirrhosis due to other causes because no quality-of-life studies for NASH cirrhosis were available to inform our model.^34^ This approach has been used in a previous cost-effectiveness analysis studying NASH.^10^ All patients who underwent surgery had a decrement in their quality of life, which was applied for 6 weeks. An additional quality-of-life decrement was applied for 6 weeks to patients who experienced major complications, and a 4-week decrement was applied after mild complications. Complications also incurred additional costs.^27^ We adjusted quality of life based on patients’ age, sex, and weight class (eTable 2 in the Supplement). Costs and utilities were discounted at an annual rate of 3%.^35^

### Outcomes

Compared with usual care, we estimated the gain in life-years, quality-adjusted life-years (QALYs), and total costs for patients undergoing ILI, SG, and GB. We assessed cost-effectiveness for each treatment strategy by estimating incremental cost-effectiveness ratios (ICERs) for patients with overweight, mild obesity, moderate obesity, and severe obesity. The ICERs were calculated on an efficiency frontier. The willingness-to-pay threshold to determine cost-effectiveness was $100 000 per QALY.

### Sensitivity Analysis

Data were collected on March 22, 2017. We conducted 1-way sensitivity analyses to examine the association of model parameter uncertainty on the cost-effectiveness results for patients in each weight class. One-way sensitivity analyses were performed for the cost-effective strategy in each weight class (ie, SG). We also performed probabilistic sensitivity analysis (PSA) to assess how model parameter uncertainty could affect our results. In PSA, all parameter values were varied simultaneously, using statistical distributions derived from the literature (Table 1). We conducted PSA using second-order sampling for 10 000 iterations for each weight class.

**Table 1.** Model Inputs: Baseline Values, Ranges, and Parameters for Distributions Used in Deterministic and Probabilistic Sensitivity Analyses

Parameter	Source	Base Case (Range or Variation)	Distribution	Parameter 1^a^	Parameter 2^b^
Age, y	Berzigotti et al,^2^ 2011	54 (34.4 to 73.6)	NA	NA	NA
Female, %	Berzigotti et al,^2^ 2011	41 (35 to 49)	β	59.122	85.078
Annual discount rate, %	Weinstein et al,^35^ 1996	3 (0 to 5)	β	8.354	270.122
Transition probabilities					
HR per 1-U increase in BMI	Berzigotti et al,^2^ 2011	1.06 (1.01 to 1.12)	Log normal	4.907	86.682
CC to DC (BMI, 27.2), %	NA	7.21 (3.21 to 10.77)	β	13.0128	167.469
CC/DC to HCC, %^10,11,12,13,14^	Mahady et al,^10^ 2012; Sanyal et al,^11^ 2006; Ratziu et al,^12^ 2002; Ascha et al,^13^ 2010; Yatsuji et al,^14^ 2009	0.69 (0.50 to 16.8)	β	1.03	147.97
CC to liver-related death, %	Sanyal et al,^11^ 2006; Hui et al,^22^ 2003	2.1 (2.0 to 4.0)	β	4.483	210.003
DC to liver-related death, %	Ratziu et al,^12^ 2002	13.0 (10.0 to 38.0)	β	0.774	5.178
HCC to liver-related death, %	Fattovich et al,^21^ 2002	42.7 (33.0 to 86.0)	β	2.14	2.87
DC to transplantation, %	Thuluvath et al,^15^ 2010; Davis et al,^16^ 2011	2.3 (1.0 to 6.2)	β	1.282	54.473
HCC to transplantation, %	Lang et al,^17^ 2009; Saab et al,^18^ 2010	4.0 (0.0 to 14.0)	β	0.59	14.16
Posttransplant to liver-related death in year 1, %	Wolfe et al,^19^ 2010	11.6 (6.0 to 42.0)	β	0.38	2.88
Posttransplant to liver-related death in year 2 or later, %^19^	Wolfe et al,^19^ 2010	4.4 (2.4 to 11.0)	β	1.59	34.51
30-Day mortality for GB, %	Mosko and Nguyen,^9^ 2011; Young et al,^23^ 2015	0.3255 (0.10 to 1.00)	β	2.000	612.491
30-Day mortality for SG, %	Mosko and Nguyen,^9^ 2011; Young et al,^23^ 2015	0.217 (0.041 to 1.14)	β	0.596	273.886
Minor complications for GB, %	Mosko and Nguyen,^9^ 2011; Young et al,^23^ 2015	7.86 (3.73 to 16.47)	β	5.311	62.255
Major complications for GB, %	Mosko and Nguyen,^9^ 2011; Young et al,^23^ 2015	5.34 (2.53 to 11.19)	β	5.477	97.095
Minor complications for SG, %	Mosko and Nguyen,^9^ 2011; Young et al,^23^ 2015	5.32 (2.52 to 11.15)	β	5.476	97.449
Major complications for SG, %	Mosko and Nguyen,^9^ 2011; Young et al,^23^ 2015	3.47 (1.65 to 7.28)	β	5.600	155.785
Health-related quality-of-life weights					
CC	Chhatwal et al,^34^ 2015	0.90 (0.81 to 0.99)	Β	37.52	4.17
DC	Chhatwal et al,^34^ 2015	0.80 (0.57 to 0.99)	β	8.50	2.12
HCC	Chhatwal et al,^34^ 2015	0.79 (0.54 to 0.99)	β	7.27	1.93
Liver transplant at year 1	Chhatwal et al,^34^ 2015	0.84 (0.77 to 0.93)	β	52.70	10.04
Liver transplant at year 2 or later	Chhatwal et al,^34^ 2015	0.93 (0.84 to 0.99)	β	27.78	2.09
Treatment-related quality-of-life weights					
Initial surgery	Campbell et al,^27^ 2010	–0.22 (–0.24 to –0.20)	β	300.96	1067.03
Minor complications	Campbell et al,^27^ 2010	–0.11 (–0.12 to –0.10)	β	369.31	2988.03
Major complications	Campbell et al,^27^ 2010	–0.36 (–0.40 to –0.32)	β	258.87	460.21
Weight-related quality-of-life weights					
BMI <30.0	Campbell et al,^27^ 2010	0.88 (0.79 to 0.97)	β	48.22	6.58
BMI 30.0-34.9	Campbell et al,^27^ 2010	0.85 (0.77 to 0.93)	β	60.27	10.64
BMI 35.0-39.9	Campbell et al,^27^ 2010	0.82 (0.74 to 0.90)	β	72.33	15.88
BMI ≥40.0	Campbell et al,^27^ 2010	0.78 (0.70 to 0.86)	β	88.40	24.93
Health state costs, 2017 $US					
CC	Saab et al,^30^ 2014; Gordon et al,^31^ 2012; McAdam-Marx et al,^32^ 2011	$5886 (±25%)	γ	61.466	0.010
DC	Saab et al,^30^ 2014; Gordon et al,^31^ 2012; McAdam-Marx et al,^32^ 2011	$41 082 (±25%)	γ	61.466	0.001
HCC	Saab et al,^30^ 2014; Gordon et al,^31^ 2012; McAdam-Marx et al,^32^ 2011	$90 344 (±25%)	γ	61.466	6.804
Liver transplant year 1	Saab et al,^30^ 2014; Gordon et al,^31^ 2012; McAdam-Marx et al,^32^ 2011	$183 279 (±25%)	γ	61.466	3.354
Liver transplant year 2 or later	Saab et al,^30^ 2014; Gordon et al,^31^ 2012; McAdam-Marx et al,^32^ 2011	$45 107 (±25%)	γ	61.466	0.001
Treatment costs, 2017 $US					
GB	Campbell et al,^27^ 2010; AHRQ,^28^ 2014	$28 734 (±25%)	γ	61.466	0.002
SG	Campbell et al,^27^ 2010; AHRQ,^28^ 2014	$23 660 (±25%)	γ	61.466	0.003
Minor complications	Campbell et al,^27^ 2010	$1414 (±25%)	γ	61.466	0.043
Major complications	Campbell et al,^27^ 2010	$46 091 (±25%)	γ	61.466	0.001
ILI in year 1	Rushing et al,^29^ 2017	$1410 (±25%)	γ	61.466	0.044
ILI in year 2	Rushing et al,^29^ 2017	$1087 (±25%)	γ	61.466	0.057
ILI in year 3	Rushing et al,^29^ 2017	$932 (±25%)	γ	61.466	0.067
ILI in year 4	Rushing et al,^29^ 2017	$750 (±25%)	γ	61.466	0.083
ILI in years 5-8	Rushing et al,^29^ 2017	$564 (±25%)	γ	61.466	0.111

Abbreviations: AHRQ, Agency for Healthcare Research and Quality; BMI, body mass index (calculated as weight in kilograms divided by height in meters squared); CC, compensated cirrhosis; DC, decompensated cirrhosis; GB, laparoscopic Roux-en-Y gastric bypass; HCC, hepatocellular carcinoma; HR, hazard ratio; ILI, intensive lifestyle intervention; NA, not applicable; SG, laparoscopic sleeve gastrectomy.

^a^ Corresponds to the α parameter for β distribution, the k (shape) parameter for γ distribution, and μ for log normal distribution.

^b^ Corresponds to the β parameter for β distribution, θ (scale) parameter for γ distribution, and α for log normal distribution.

## Results

### Base-Case Results

All weight loss strategies involved a gain in life-years and QALYs in patients with compensated cirrhosis (41% female and 59% male; base case age, 54 years). Compared with usual care, SG was associated with an increase in QALYs of 0.263 to 1.180 (bounds of ranges represent overweight to severe obesity); GB, 0.263 to 1.207; and ILI, 0.004 to 0.216. Sleeve gastrectomy was also associated with an increase in life-years of 0.693 to 1.930; GB, 0.694 to 1.947; and ILI, 0.012 to 0.114 (Table 2). With usual care, expected life-years in overweight, mild obesity, moderate obesity, and severe obesity were 12.939, 11.949, 10.976, and 10.095, respectively. These results are consistent with prospective studies that found median survival of greater than 12 years in patients with compensated cirrhosis.^36^ With usual care, QALY in overweight was 6.418; mild obesity, 5.790; moderate obesity, 5.186; and severe obesity, 4.577. For patients with obesity (mild, moderate, and severe), GB involved the greatest increase in life-years and QALYs, followed by SG and then ILI. In contrast, for overweight patients, GB had the largest increase in life-years; however, SG had the largest increase in QALYs, followed very closely by GB and then by ILI. Note that these differences between GB and SG for overweight patients were very small. When rounded to the nearest thousandth, SG and GB both involved an equal number of QALYs, and GB involved 0.001 additional life-years compared with SG.

**Table 2.** Results of Cost-effectiveness Analyses^a^

Strategy	Cost, 2017 $US	Incremental Cost, 2017 $US	QALYs	Incremental QALYs	Life-Years	Incremental Life-Years	ICER, $/QALY
Severe obesity (BMI ≥40.0)							
Usual care	214 412	NA	4.577	NA	10.095	NA	NA
ILI	223 087	934	4.793	−0.964	10.209	−1.815	Absolutely dominated
SG	222 153	7741	5.757	1.179	12.025	1.930	6563
GB	228 369	6216	5.784	0.027	12.042	0.017	229 919
Moderate obesity (BMI 35.0-39.9)							
Usual care	206 809	NA	5.186	NA	10.976	NA	NA
ILI	214 953	8145	5.259	0.073	11.063	0.087	Extendedly dominated
SG	216 075	1122	6.088	0.829	12.593	1.530	10 274
GB	222 174	6099	6.106	0.019	12.604	0.011	329 002
Mild obesity (BMI 30.0-34.9)							
Usual care	197 486	NA	5.790	NA	11.949	NA	NA
ILI	205 128	7642	5.809	0.020	12.001	0.052	Extendedly dominated
SG	209 976	4848	6.457	0.648	13.131	1.130	18 716
GB	215 990	6014	6.458	0.002	13.137	0.006	3 667 701
Overweight (BMI 25.0-29.9)							
Usual care	186 264	NA	6.418	NA	12.939	NA	NA
ILI	193 506	7242	6.422	0.004	12.951	0.012	Extendedly dominated
SG	203 660	10 153	6.681	0.258	13.632	0.681	66 119
GB	209 606	5946	6.681	0	13.633	0.001	Absolutely dominated

Abbreviations: BMI, body mass index (calculated as weight in kilograms divided by height in meters squared); GB, laparoscopic Roux-en-Y gastric bypass; ICER, incremental cost-effectiveness ratio; ILI, intensive lifestyle intervention; NA, not applicable; QALYs, quality-adjusted life-years; SG, laparoscopic sleeve gastrectomy.

^a^ An extendedly dominated strategy has an ICER that is higher than that of the next most effective strategy. An absolutely dominated strategy is more expensive and less effective than other strategies. Note that the ILI strategy has negative incremental QALYs and life-years for the severe obesity category; as the ILI strategy was absolutely dominated, the incremental QALYs, life-years, and costs for this group are calculated against the SG strategy.

Cost-effectiveness analysis found that SG was the optimal strategy in patients with NASH cirrhosis in all weight categories. Compared with the next nondominated strategy, the ICER for SG was $66 119 per QALY in overweight, $18 716 per QALY in mild obesity, $10 274 per QALY in moderate obesity, and $6563 per QALY in severe obesity (Table 2). Although GB involved a greater increase in QALYs than SG in obese patients, GB was also more expensive, and the ICER for GB ($229 919 per QALY for severe, $329 002 per QALY for moderate, and $3 667 701 per QALY for mild obesity) exceeded the commonly accepted willingness-to-pay threshold of $100 000 per QALY.

A threshold analysis on the procedure cost of GB found that for GB to be cost-effective, the cost of the surgery must be decreased from its baseline value of $28 734 by $4889 for mild obesity, by $3189 for moderate obesity, and by $2289 for severe obesity (Table 3). In overweight patients, GB involved fewer QALYs than SG, and thus decreasing the cost of surgery would not result in cost-effectiveness.

**Table 3.** Threshold Analysis: Procedure Cost That Could Make GB Cost-effective^a^

Patient Profile	Reduced Cost of GB at Which GB Is the Most Cost-effective Intervention $	Cost Reduction Relative to Baseline Cost of GB, 2017 $US
Severe obesity (BMI ≥40.0)	25 965	2289
Moderate obesity (BMI 35.0-39.9)	25 065	3189
Mild obesity (BMI 30.0-34.9)	23 365	4889

Abbreviations: BMI, body mass index (calculated as weight in kilograms divided by height in meters squared); GB, laparoscopic Roux-en-Y gastric bypass.

^a^ Baseline cost of GB was $28 734. Overweight is not included because GB was absolutely dominated for this BMI class (ie, GB resulted in fewer quality-adjusted life-years than sleeve gastrectomy).

### Sensitivity Analysis

We conducted 1-way sensitivity analysis only on the cost-effective interventions identified in the base case analyses (ie, SG). eFigures 1 to 4 in the Supplement show the 10 model parameters for each weight class that had the largest association with the ICER, using tornado diagrams. We found that in patients with mild, moderate, or severe obesity, SG remained cost-effective despite variations in all model inputs. In overweight patients, the ICER for SG remained below the willingness-to-pay threshold ($100 000/QALY), despite modifications to all model inputs except for age and the hazard ratio for decompensation associated with a 1-U increase in BMI. The ICER rose above $100 000 per QALY (vs usual care) when age was varied to its high extreme (73.6 years) and when the hazard ratio for decompensation associated with a 1-U increase in BMI was varied to its low extreme (1.01).

In the PSA, model parameters were varied simultaneously 10 000 times, and cost-effectiveness was calculated. According to our PSA results, SG was the most cost-effective option for patients with overweight or obesity in most iterations. In patients with severe obesity, SG was the optimal strategy in 79% of iterations, using a willingness-to-pay threshold of $100 000 per QALY (eFigure 5 in the Supplement). In those with moderate obesity, SG was the most cost-effective option in 64% of iterations (eFigure 6 in the Supplement). In patients with mild obesity, it was the most cost-effective option in 76% of iterations (eFigure 7 in the Supplement). In the overweight population, SG was the optimal strategy in 53% of model iterations (eFigure 8 in the Supplement).

## Discussion

In this modeling-based study, we examined whether the benefits of bariatric surgery outweighed the heightened risks of mortality and complications in surgical candidates with NASH cirrhosis. Our results projected that surgery would outperform usual care and ILI across all weight classes assessed, including overweight, in terms of life expectancy and QALY. Furthermore, we found that bariatric surgery was cost-effective for patients with obesity (mild, moderate, and severe) and those in the overweight range.

Although studies demonstrate that decompensation is more likely at higher BMIs^2^ and that weight loss leads to decreased portal hypertension in patients with cirrhosis and obesity,^5^ less is known regarding the effect of bariatric surgery on clinical outcomes in NASH cirrhosis.^7,8^ In a previous analysis, Klebanoff et al^37^ examined the long-term cost-effectiveness of bariatric surgery in patients with NASH and varying degrees of fibrosis, but that study did not include patients with NASH and cirrhosis. However, given the continued rise in the prevalence of NASH cirrhosis,^38^ we should explore the potential role of weight loss in treating this disease. Therefore, our analysis underscores a real and urgent need for clinical studies of bariatric surgery and other weight loss therapies in NASH cirrhosis.

For patients with obesity, bariatric surgery remained cost-effective in our 1-way sensitivity analyses, which included varying the probability of surgical mortality and complications. These findings are important because the weighing of risks and benefits of bariatric surgery in the setting of cirrhosis may not appear obvious to patients or physicians. Surgery may involve a 3-fold increased risk of death in patients with compensated cirrhosis, compared with those without cirrhosis.^9^ In addition, individuals with cirrhosis have a diminished life expectancy, and thus may not derive long-term health benefits from surgery, including improvements in comorbidities such as hypertension, dyslipidemia, and diabetes.^39,40^ Despite these considerations, our analysis suggests that the benefits of surgery may outweigh the risks. In sensitivity analyses, surgery remained cost-effective in patients with obesity, including those with mild obesity (BMI, 30.0-35.0), even when we assumed a small benefit of weight loss on liver disease progression and an increase in the the risk of surgical mortality. In addition, a recent analysis found no association between compensated cirrhosis and increased mortality in patients undergoing bariatric surgery, suggesting that our analysis may even overstate the dangers of bariatric surgery.^41^ Our findings are consistent with the available evidence, suggesting that bariatric surgery offers an acceptably low risk in patients with compensated cirrhosis, potentially even in those with portal hypertension.^42,43^

To date, no clinical studies have compared outcomes of SG and GB in patients with NASH cirrhosis. We chose to include both procedures in our model, because they are the 2 most common bariatric procedures performed in the United States.^44^ Of the treatment options in this analysis, GB involved the largest increase in life expectancy and quality of life for patients with obesity, but SG was the most cost-effective. Although GB involved a slightly greater increase in QALYs, it was also marginally more expensive than SG. Our findings suggest that the slightly superior weight loss attributable to GB might not be worth the higher cost of the procedure. In addition to the extra cost of GB, it also entails a higher risk of complications, which may limit its cost-effectiveness. Data from patients without cirrhosis show that GB induces more weight loss and more successful resolution of comorbidities compared with SG, but also involves a higher rate of deep wound infections, serious morbidity, and 30-day reoperation.^23^ Despite the overall lower rate of complications for SG, it is associated with an increased risk of portal vein thrombosis, a rare but serious complication with mortality exceeding 40% among affected patients.^45^ Although this risk poses an important clinical consideration, patients and physicians are increasingly opting for SG, owing to various potential advantages. For example, SG preserves endoscopic access to the gastric tube in the event of variceal bleeding, as well as access to the biliary system.^46^ While our model is informed by limited data and should not be used to guide selection of a particular bariatric procedure, our findings support the idea that SG may provide a more favorable combination of risk, benefit, and cost compared with GB. Given the rapidly increasing popularity of SG at the expense of GB, our results may reflect a growing conventional wisdom concerning these procedures among bariatric surgeons.

### Strengths and Limitations

Our study has several strengths. This model is, to our knowledge, the first cost-effectiveness analysis of bariatric surgery and lifestyle intervention among patients with NASH cirrhosis. In addition, although 1 previous modeling study explored weight loss in patients with cirrhosis,^47^ it did not assess cost-effectiveness and did not include SG, which is now the most popular bariatric procedure in the United States.^48^ Compared with prior analyses, ours also incorporated not only individuals with severe obesity, but also those with lower baseline BMI.

As with any simulation model, our analysis must be viewed in the context of some limitations. First, we used weight loss data from studies of patients without cirrhosis; weight loss after bariatric surgery may differ between patients with and without cirrhosis. This limitation is not major, because the available data suggest excellent weight loss outcomes for patients with and without cirrhosis.^7^ We also extrapolated long-term trends in BMI using data from the Swedish Obese Subjects study,^26^ given a lack of long-term data surrounding trends in weight after SG and GB. In addition, we assumed that weight loss would slow the progression of compensated cirrhosis to decompensated cirrhosis. This decision gains support from an analysis that described an increased risk of decompensation associated with high BMI,^2^ as well as a trial that found decreased portal hypertension after weight loss.^5^ Evidence also suggests that bariatric surgery induces resolution of noncirrhotic NASH,^49^ and some limited data show that surgery may even cause regression of fibrosis in patients with cirrhosis.^8,50^ Future studies that explore the effects of bariatric surgery in this population will confirm or refute our modeling assumptions. We also assumed that weight loss would benefit patients in our model by leading to a decrease in background mortality, a decrease in the probability of decompensation, and an increase in weight-related quality of life. Thus, we may have overestimated the benefit of surgery on survival and quality of life. However, we also did not explicitly model other comorbidities that resolve or improve with weight loss, such as diabetes, hypertension, and dyslipidemia, which may counterbalance any potential overestimation of clinical benefit. In addition, to inform the effect of weight loss on the probability of decompensation, our analysis used data from a study that included a large portion of patients with cirrhosis secondary to viral hepatitis or alcohol use.^2^ Given the association between NASH and obesity, we suspect that weight loss might have an even greater benefit in individuals with NASH. Accordingly, our estimates of the benefits of bariatric surgery in this population may be conservative.

Our results for patients with a BMI of less than 35.0 should also be viewed with some caution. The available evidence suggests that bariatric surgery is safe and does not lead to excessive weight loss in patients with mild obesity. Based on the current literature, the American Society for Metabolic and Bariatric Surgery^51^ and the International Federation for the Surgery of Obesity and Metabolic Disorders^52^ recommend bariatric surgery as an option for suitable patients with a BMI of 30.0 to 35.0. For patients with a BMI of less than 30.0, scant data are available to guide clinical decision making. The lack of data to inform modeling for patients with a BMI of less than 30.0 makes our analysis of the overweight patients an exploratory one, although we can reasonably expect similar beneficial results of surgery in the lower BMI group.

## Conclusions

Our results underscore the promise that bariatric procedures may hold for patients with NASH cirrhosis, including those with lower BMI. Our analysis affirms that the benefits of surgery likely outweigh the risks in otherwise eligible individuals, and surgery could be a cost-effective intervention. Given the increasing burden of NASH cirrhosis and the lack of effective treatments, it would appear that future studies must explore the effects of bariatric surgery on disease progression in individuals with NASH cirrhosis.

## Notes

Supplement. **eTable 1.** Percentage of Excess Weight Loss After Bariatric Surgery and Lifestyle Intervention **eTable 2.** Health-Related Quality-of-Life Utilities of the United States Population **eFigure 1.** One-Way Sensitivity Analysis for SG in Severe Obesity **eFigure 2.** One-Way Sensitivity Analysis for SG in Moderate Obesity **eFigure 3.** One-Way Sensitivity Analysis for SG in Mild Obesity **eFigure 4.** One-Way Sensitivity Analysis for SG in Overweight **eFigure 5.** Probabilistic Sensitivity Analysis for Severe Obesity **eFigure 6.** Probabilistic Sensitivity Analysis for Moderate Obesity **eFigure 7.** Probabilistic Sensitivity Analysis for Mild Obesity **eFigure 8.** Probabilistic Sensitivity Analysis for Overweight **eReferences.**
