# Supplementary material for: Cost-effectiveness Analysis of Bariatric Surgery for Patients With Nonalcoholic Steatohepatitis Cirrhosis
Source: JAMA Netw Open. 2019 Feb 22;2(2):e190047. doi: 10.1001/jamanetworkopen.2019.0047 (PMC6484583; doi:10.1001/jamanetworkopen.2019.0047)
Supplement: Supplement. — eTable 1. Percentage of Excess Weight Loss After Bariatric Surgery and Lifestyle Intervention eTable 2. Health-Related Quality-of-Life Utilities of the United States Population eFigure 1. One-Way Sensitivity Analysis for SG in Severe Obesity eFigure 2. One-Way Sensitivity Analysis for SG in Moderate Obesity eFigure 3. One-Way Sensitivity Analysis for SG in Mild Obesity eFigure 4. One-Way Sensitivity Analysis for SG in Overweight eFigure 5. Probabilistic Sensitivity Analysis for Severe Obesity eFigure 6. Probabilistic Sensitivity Analysis for Moderate Obesity eFigure 7. Probabilistic Sensitivity Analysis for Mild Obesity eFigure 8. Probabilistic Sensitivity Analysis for Overweight eReferences. [file jamanetwopen-2-e190047-s001.pdf]

Supplementary Online Content

Klebanoff MJ, Corey KE, Samur S, et al. Cost-effectiveness analysis of bariatric surgery for patients with nonalcoholic steatohepatitis cirrhosis. *JAMA Netw Open*. 2019;2(2):e190047. doi:10.1001/jamanetworkopen.2019.0047

eTable 1. Percentage of Excess Weight Loss After Bariatric Surgery and Lifestyle Intervention

eTable 2. Health-Related Quality-of-Life Utilities of the United States Population

eFigure 1. One-Way Sensitivity Analysis for SG in Severe Obesity

eFigure 2. One-Way Sensitivity Analysis for SG in Moderate Obesity

eFigure 3. One-Way Sensitivity Analysis for SG in Mild Obesity

eFigure 4. One-Way Sensitivity Analysis for SG in Overweight

eFigure 5. Probabilistic Sensitivity Analysis for Severe Obesity

eFigure 6. Probabilistic Sensitivity Analysis for Moderate Obesity

eFigure 7. Probabilistic Sensitivity Analysis for Mild Obesity

eFigure 8. Probabilistic Sensitivity Analysis for Overweight

eReferences.

This supplementary material has been provided by the authors to give readers additional information about their work.

© 2019 Klebanoff MJ et al. *JAMA Network Open*.

eTable 1. Percentage of Excess Weight Loss After Bariatric Surgery and Lifestyle Intervention

	GB [1,2]		SG [1,2]		ILI [3]	
**Year**	**Base-Case**	**Range**	**Base-Case**	**Range**	**Base-Case**	**Range**
0	0.00%	0– 0.00%	0.00%	0– 0.00%	0.00%	0– 0.00%
1	-72.32%	-64.60%– -80.04%	-69.70%	-41.09%– -98.32%	-28.14%	-27.50%– -28.78%
2	-71.02%	-70.84%– -71.44%	-68.45%	-68.27%– -68.85%	-20.77%	-20.08%– -21.45%
3	-66.08%	-65.33%– -67.15%	-63.68%	-62.96%– -64.72%	-16.27%	-15.58%– -16.95%
4	-63.74%	-62.91%– -64.51%	-61.43%	-60.63%– -62.17%	-14.60%	-13.93%– -15.26%
5	-61.33%	-60.29%– -62.30%	-59.11%	-58.10%– -60.05%	-13.28%	-12.59%– -13.96%
6	-58.92%	-57.67%– -60.10%	-56.79%	-55.58%– -57.92%	-13.90%	-13.24%– -14.56%
7	-56.84%	-55.52%– -58.15%	-54.78%	-53.51%– -56.04%	-13.29%	-12.60%– -13.97%
8	-54.76%	-53.37%– -56.19%	-52.78%	-51.43%– -54.16%	-15.59%	-14.95%– -16.23%
9	-53.59%	-51.95%– -55.18%	-51.65%	-50.07%– -53.19%	-15.59%	-14.95%– -16.23%
10	-52.42%	-50.54%– -54.18%	-50.52%	-48.71%– -52.21%	-15.59%	-14.95%– -16.23%
11	-53.80%	-50.92%– -56.52%	-51.85%	-49.08%– -54.47%	-15.59%	-14.95%– -16.23%
12	-55.18%	-51.30%– -58.86%	-53.18%	-49.44%– -56.73%	-15.59%	-14.95%– -16.23%
13	-56.56%	-51.67%– -61.21%	-54.51%	-49.80%– -58.99%	-15.59%	-14.95%– -16.23%
14	-57.93%	-52.05%– -63.55%	-55.84%	-50.16%– -61.25%	-15.59%	-14.95%– -16.23%
15	-59.31%	-52.43%– -65.89%	-57.16%	-50.53%– -63.51%	-15.59%	-14.95%– -16.23%
16	-59.05%	-51.43%– -66.30%	-56.91%	-49.57%– -63.90%	-15.59%	-14.95%– -16.23%
17	-58.79%	-50.44%– -66.70%	-56.66%	-48.61%– -64.28%	-15.59%	-14.95%– -16.23%
18	-58.53%	-49.44%– -67.10%	-56.41%	-47.65%– -64.67%	-15.59%	-14.95%– -16.23%
19	-58.27%	-48.45%– -67.51%	-56.16%	-46.69%– -65.06%	-15.59%	-14.95%– -16.23%
20	-58.01%	-47.45%– -67.91%	-55.91%	-45.73%– -65.45%	-15.59%	-14.95%– -16.23%

Negative values indicate weight loss, whereas positive values indicate weight gain. Ranges were used in one-way sensitivity analysis. β distributions were used for all weight loss values in probabilistic sensitivity analysis.

For patients who underwent laparoscopic Roux-en-Y gastric bypass (GB) or laparoscopic sleeve gastrectomy (SG), the weight loss one year after surgery was based on a recent meta-analysis.[1] In subsequent years, maintenance of weight loss after bariatric surgery was based on data from the Swedish Obese Subjects (SOS) study.[2] Weight loss in the intensive lifestyle intervention (ILI) strategy was derived from the Look AHEAD (Action for Health in Diabetes) trial.[3] After year 8, body mass index (BMI) remained stable in the ILI strategy. In the usual care strategy, patients remained at their initial BMI throughout their lifetime.

GB, laparoscopic Roux-en-Y gastric bypass; SG, laparoscopic sleeve gastrectomy; ILI, intensive lifestyle intervention

© 2019 Klebanoff MJ et al. *JAMA Network Open.*

eTable 2. Health-Related Quality-of-Life Utilities of the United States Population

Age Group	Male	Female
**20–29**	0.928	0.913
**30–39**	0.918	0.893
**40–49**	0.887	0.863
**50–59**	0.861	0.837
**60–69**	0.840	0.811
**70–79**	0.802	0.771
**80–89**	0.782	0.724

The age/sex-specific utilities in **eTable 2** were used to adjust the quality-of-life values assigned during each year to patients in the model. These utilities were multiplied with the liver disease utility (i.e., the utility for compensated cirrhosis, decompensated cirrhosis, hepatocellular carcinoma, or post-transplant health states) and the weight-related utility to determine each patient's overall utility during each year of the simulation. Source: Hanmer et al.[4]

© 2019 Klebanoff MJ et al. *JAMA Network Open.*

eFigure 1. One-Way Sensitivity Analysis for SG in Severe Obesity

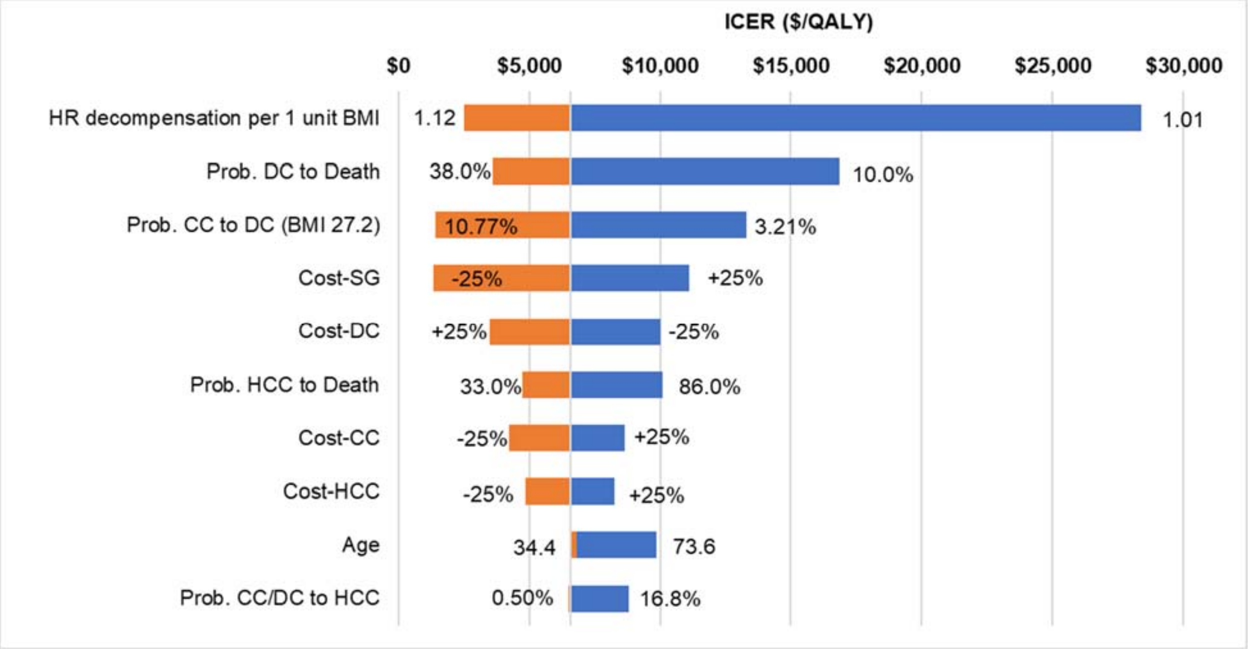

eFigure 1 shows the results of one-way sensitivity analyses performed for SG (versus usual care) in patients with severe obesity. One-way sensitivity analysis involves adjusting the value of one model parameter at a time in order to assess the impact on study outcomes. This figure includes the ten parameters that led to the largest effect on ICER values when modified. The numbers on either side of the bars indicate the extreme parameter values that led to the resulting ICER shown in the figure. This figure is centered around the base-case ICER of $6,563/QALY. High and low parameter values can be found in **Table 1** and **eTable 1**.

ICER, incremental cost-effectiveness ratio; QALY, quality-adjusted life year; HR, hazard ratio; BMI, body mass index; Prob., probability; DC, decompensated cirrhosis; CC, compensated cirrhosis; SG, laparoscopic sleeve gastrectomy; HCC, hepatocellular carcinoma

© 2019 Klebanoff MJ et al. *JAMA Network Open.*

eFigure 2. One-Way Sensitivity Analysis for SG in Moderate Obesity

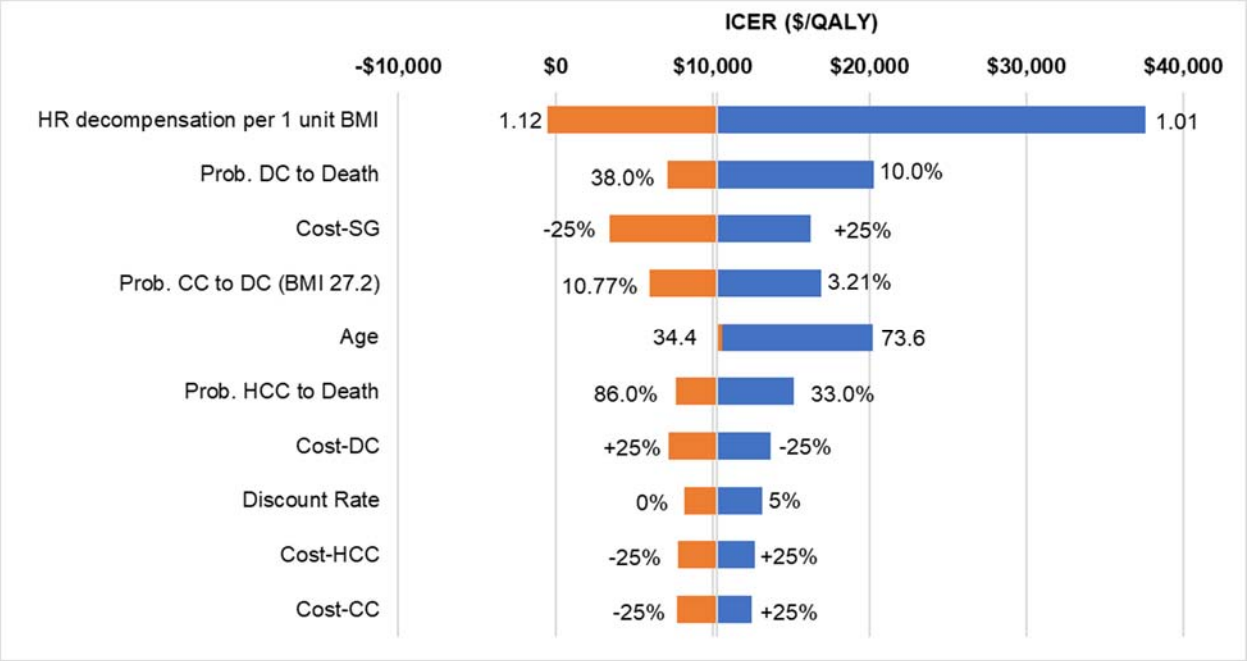

eFigure 2 shows the results of one-way sensitivity analyses performed for SG (versus usual care) in patients with moderate obesity. One-way sensitivity analysis involves adjusting the value of one model parameter at a time in order to assess the impact on study outcomes. This figure includes the ten parameters that led to the largest effect on ICER values when modified. The numbers on either side of the bars indicate the extreme parameter values that led to the resulting ICER shown in the figure. This figure is centered around the base-case ICER of $10,274/QALY. High and low parameter values can be found in **Table 1** and **eTable 1**.

ICER, incremental cost-effectiveness ratio; QALY, quality-adjusted life year; HR, hazard ratio; BMI, body mass index; Prob., probability; DC, decompensated cirrhosis; SG, laparoscopic sleeve gastrectomy; CC, compensated cirrhosis; HCC, hepatocellular carcinoma

© 2019 Klebanoff MJ et al. *JAMA Network Open.*

eFigure 3. One-Way Sensitivity Analysis for SG in Mild Obesity

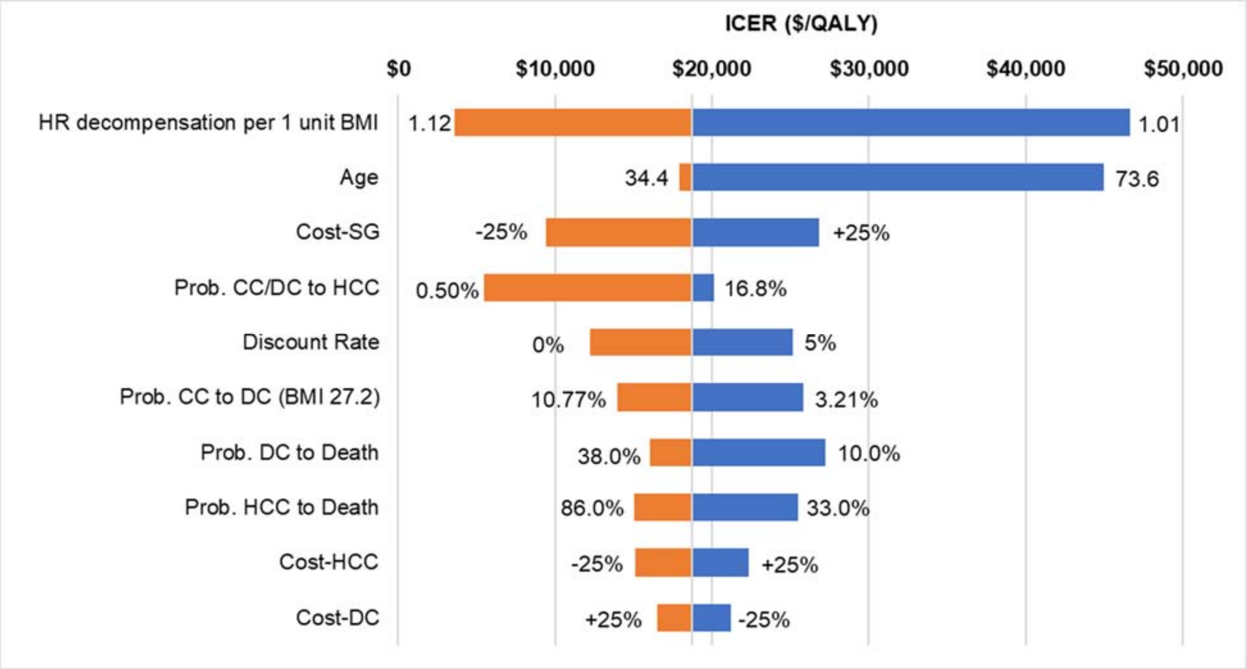

eFigure 3 shows the results of one-way sensitivity analyses performed for SG (versus usual care) in patients with mild obesity. One-way sensitivity analysis involves adjusting the value of one model parameter at a time in order to assess the impact on study outcomes. This figure includes the ten parameters that led to the largest effect on ICER values when modified. The numbers on either side of the bars indicate the extreme parameter values that led to the resulting ICER shown in the figure. This figure is centered around the base-case ICER of $18,716/QALY. High and low parameter values can be found in **Table 1** and **eTable 1**.

ICER, incremental cost-effectiveness ratio; QALY, quality-adjusted life year; BMI, body mass index; SG, laparoscopic sleeve gastrectomy; Prob., probability; CC, compensated cirrhosis; DC, decompensated cirrhosis; HCC, hepatocellular carcinoma

© 2019 Klebanoff MJ et al. *JAMA Network Open.*

eFigure 4. One-Way Sensitivity Analysis for SG in Overweight

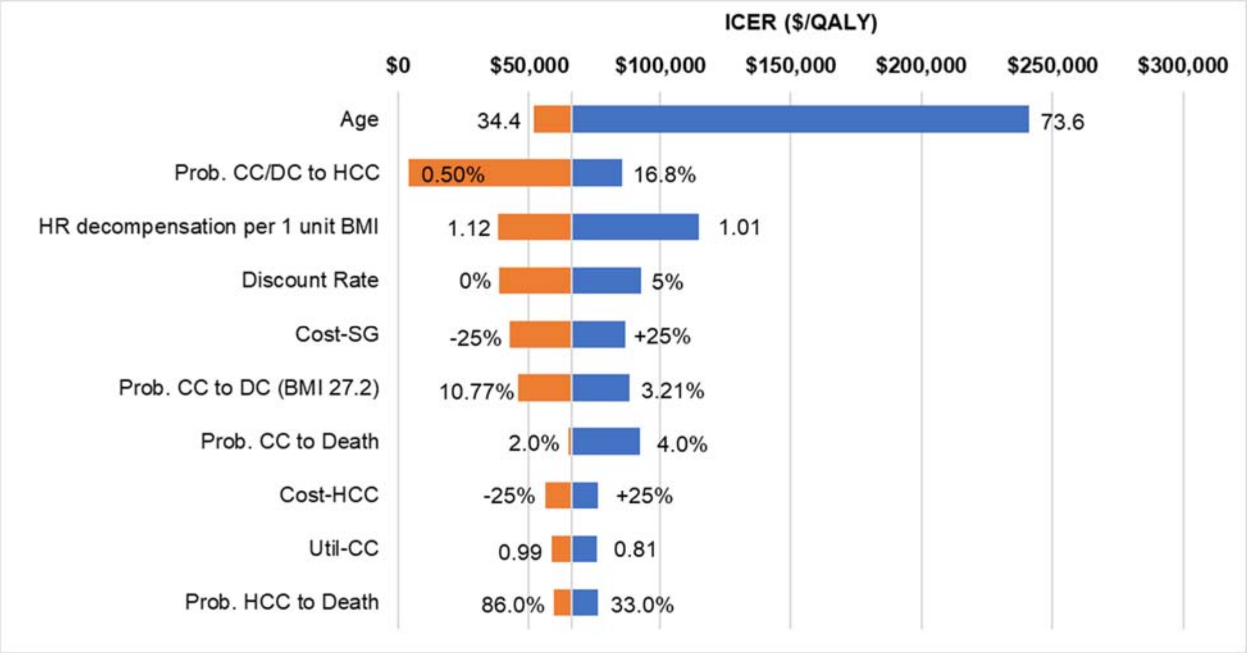

eFigure 4 shows the results of one-way sensitivity analyses performed for SG (versus usual care) in patients with overweight. One-way sensitivity analysis involves adjusting the value of one model parameter at a time in order to assess the impact on study outcomes. This figure includes the ten parameters that led to the largest effect on ICER values when modified. The numbers on either side of the bars indicate the extreme parameter values that led to the resulting ICER shown in the figure. This figure is centered around the base-case ICER of $66,119/QALY. High and low parameter values can be found in **Table 1** and **eTable 1**.

ICER, incremental cost-effectiveness ratio; QALY, quality-adjusted life year; Prob., probability; CC, compensated cirrhosis; DC, decompensated cirrhosis; HCC, hepatocellular carcinoma; HR, hazard ratio; BMI, body mass index; SG, laparoscopic sleeve gastrectomy; Util, utility

© 2019 Klebanoff MJ et al. *JAMA Network Open.*

eFigure 5. Probabilistic Sensitivity Analysis for Severe Obesity

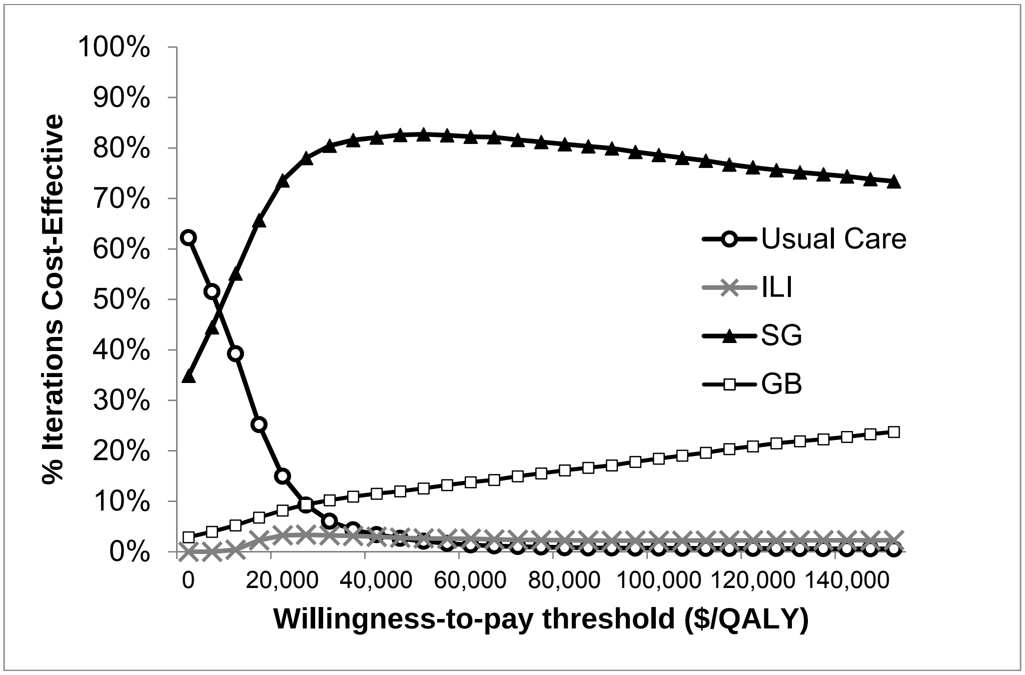

Probabilistic sensitivity analysis was performed for patients with severe obesity (i.e., BMI>40). The model was run using second-order sampling for 10,000 iterations; the percent of these times that a treatment strategy was cost-effective, at varying willingness-to-pay thresholds, is shown in this figure.

ILI, intensive lifestyle intervention; SG, laparoscopic sleeve gastrectomy; GB, laparoscopic Roux-en-Y gastric bypass; QALY, quality-adjusted life year

© 2019 Klebanoff MJ et al. *JAMA Network Open.*

eFigure 6. Probabilistic Sensitivity Analysis for Moderate Obesity

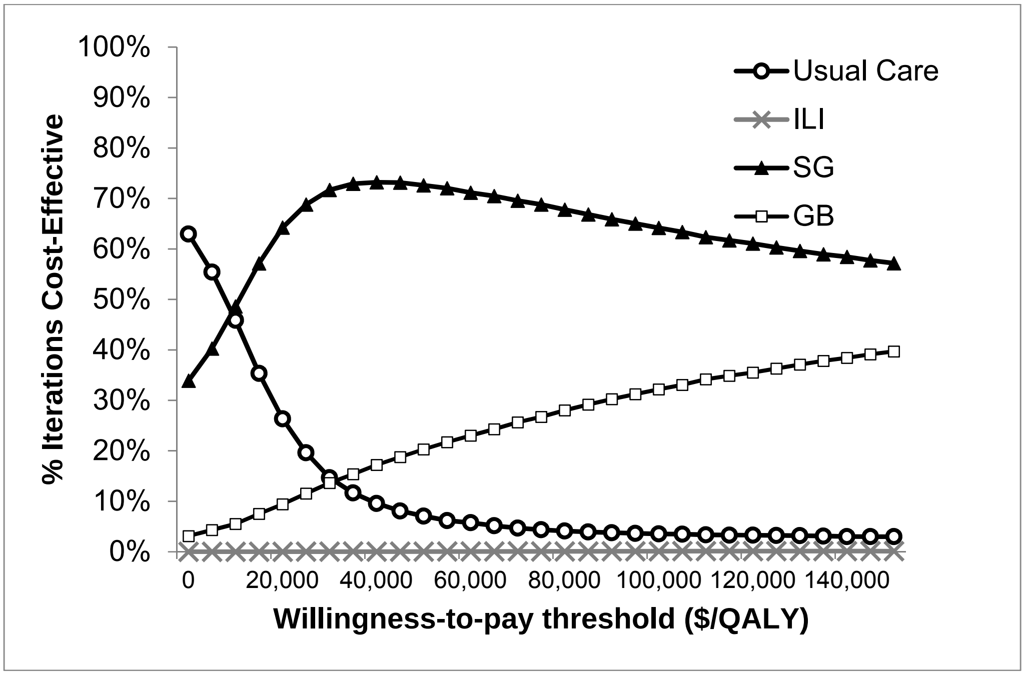

Probabilistic sensitivity analysis was performed for patients with moderate obesity (i.e., BMI 35 – 40). The model was run using second-order sampling for 10,000 iterations; the percent of these times that a treatment strategy was cost-effective, at varying willingness-to-pay thresholds, is shown in this figure.

ILI, intensive lifestyle intervention; SG, laparoscopic sleeve gastrectomy; GB, laparoscopic Roux-en-Y gastric bypass; QALY, quality-adjusted life year

© 2019 Klebanoff MJ et al. *JAMA Network Open.*

eFigure 7. Probabilistic Sensitivity Analysis for Mild Obesity

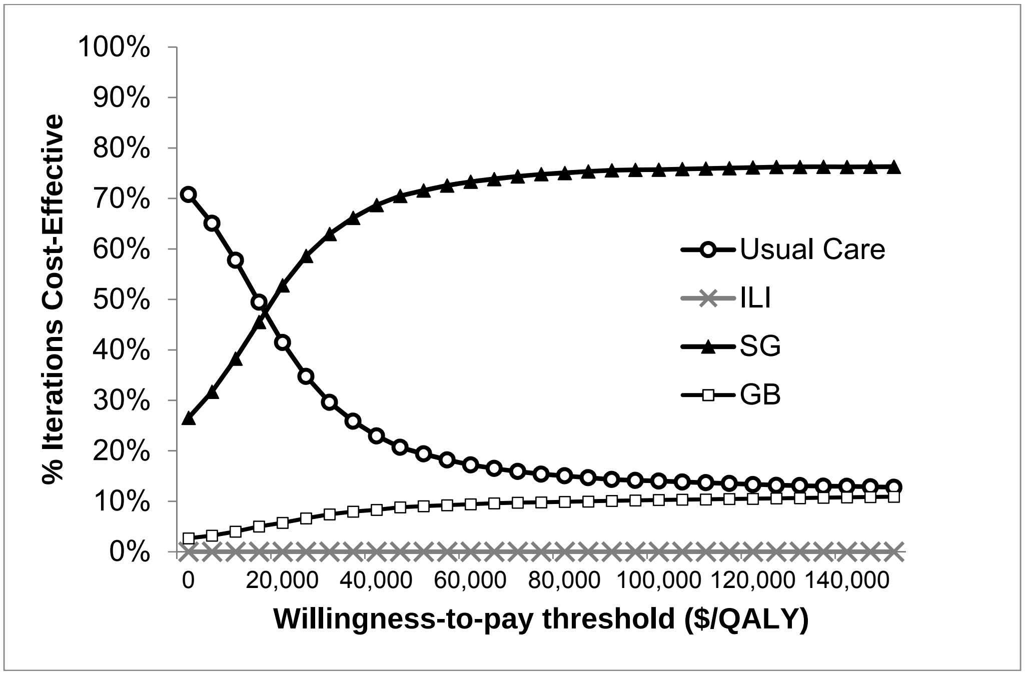

Probabilistic sensitivity analysis was performed for patients with mild obesity (i.e., BMI 30 – 35). The model was run using second-order sampling for 10,000 iterations; the percent of these times that a treatment strategy was cost-effective, at varying willingness-to-pay thresholds, is shown in this figure.

ILI, intensive lifestyle intervention; SG, laparoscopic sleeve gastrectomy; GB, laparoscopic Roux-en-Y gastric bypass; QALY, quality-adjusted life year

© 2019 Klebanoff MJ et al. *JAMA Network Open.*

eFigure 8. Probabilistic Sensitivity Analysis for Overweight

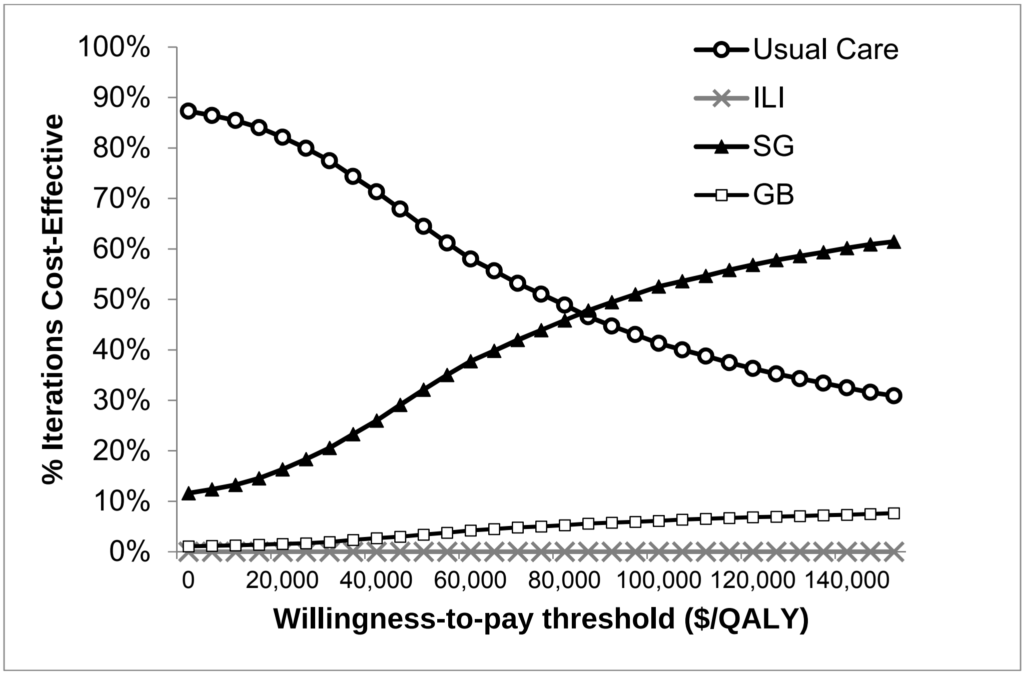

Probabilistic sensitivity analysis was performed for patients with overweight (i.e., BMI 25 – 30). The model was run using second-order sampling for 10,000 iterations; the percent of these times that a treatment strategy was cost-effective, at varying willingness-to-pay thresholds, is shown in this figure.

ILI, intensive lifestyle intervention; SG, laparoscopic sleeve gastrectomy; GB, laparoscopic Roux-en-Y gastric bypass; QALY, quality-adjusted life year

© 2019 Klebanoff MJ et al. *JAMA Network Open.*

eReferences

1. Chang SH, Stoll CR, Song J, Varela JE, Eagon CJ, Colditz GA. The effectiveness and risks of bariatric surgery: an updated systematic review and meta-analysis, 2003-2012. *JAMA Surg.* 2014;149(3):275-287.
2. Sjostrom L, Peltonen M, Jacobson P, et al. Bariatric surgery and long-term cardiovascular events. *JAMA.* 2012;307(1):56-65.
3. Look ARG. Eight-year weight losses with an intensive lifestyle intervention: the look AHEAD study. *Obesity (Silver Spring).* 2014;22(1):5-13.
4. Hanmer J, Lawrence WF, Anderson JP, Kaplan RM, Fryback DG. Report of nationally representative values for the noninstitutionalized US adult population for 7 health-related quality-of-life scores. *Medical decision making : an international journal of the Society for Medical Decision Making.* 2006;26(4):391-400.

© 2019 Klebanoff MJ et al. *JAMA Network Open.*
